# *In silico* evaluation of favipiravir-associated potential new drugs against polymerase enzyme of SARS-CoV-2

**DOI:** 10.1016/j.heliyon.2024.e38479

**Published:** 2024-09-26

**Authors:** Saira, Khalid Khan, Asad Khan, Ateeq Khan, Tanzeel Shah, Nasir Ahmad, Haroon ur Rashid, Muhammad Zahoor, Riaz Ullah, Ahmed Bari, Muhammad Naveed Umar

**Affiliations:** a Department of Chemistry, Islamia College Peshawar, Khyber Pakhtunkhwa, 25120, Pakistan; b Institute of Basic Medical Sciences, Khyber Medical University, Peshawar, Khyber Pakhtunkhwa, 25120, Pakistan; c Center of Chemical, Pharmaceutical and Food Sciences, Federal University of Pelotas, Pelotas, RS, Brazil; d Department of Biochemistry, University of Malakand at Chakdara, Dir Lower, Khyber Pakhtunkhwa, Pakistan; e Department of Pharmacognosy, College of Pharmacy, King Saud University, Riyadh, 11451, Saudi Arabia; f Department of Pharmaceutical Chemistry, College of Pharmacy King Saud University, Riyadh, Saudi Arabia; g Department of Chemistry, University of Liverpool, UK

**Keywords:** SARS-CoV-2, RdRp, Favipiravir's derivatives, Bioinformatics

## Abstract

Millions of lives have been lost to the deadly SARS-CoV-2 virus. Vaccines and antiviral drugs are essential scientific tools in combating viral infections. This *in silico* study focused on the RdRp inhibitor favipiravir, exploring new analogs by substituting the fluorine atom on the pyrazine ring with both homocyclic and heterocyclic moieties. Initially, ADME and toxicity properties were assessed using SwissADME and ProTox-II online tools. Ligands **L6** and **L7** exhibited high bioavailability and drug-likeness compared to favipiravir. Subsequently, all new analogs were docked into the RdRp active site using AutoDock Vina, demonstrating high affinity compared to favipiravir. Based on optimal ADMET profiles and docking scores, ligands **L4**, **L6**, and **L7** underwent 200 ns MDS using the CHAARM 36 force field in NAMD software to validate docking results. Various trajectory analyses, including RMSD, RMSF, histograms, total number of contacts, and ligand properties, were conducted to gain insights into the interaction patterns between ligands and RdRp. All protein-ligand complexes exhibited greater stability than favipiravir throughout simulations period. This theoretical study suggests that ligands **L6** and **L7** could serve as lead candidates for RdRp inhibition. Cell-Based SARS-CoV-2 RdRp Activity Assay is recommended to validate these *in silico* findings.

## 1 Introduction

Coronaviruses induce respiratory infections in mammals such as bats, masked palm civets, and camels, as well as in avian species [1,2]. Tissue tropism and symptoms of coronavirus infections exhibit variability across diverse host species [3]. In humans, coronavirus infections can range from asymptomatic to presenting with symptoms such as fever, cough, dyspnea, and gastrointestinal manifestations [4,5]. Particularly in elderly and immunocompromised individuals, coronavirus infections may progress to severe pneumonia, occasionally culminating in fatal outcomes [6]. Three major epidemics have been reported to date, with the most recent occurring in Wuhan, a city in China's Hubei province, in December 2019 [7,8]. The World Health Organization (WHO) declared a Public Health Emergency of International Concern on January 30, 2020, due to a new, deadly virus causing severe respiratory illness that rapidly spread worldwide [9]. Subsequently, WHO declared the COVID-19 a pendemic on March 11, 2020 [10]. As of July 2024, the severe acute respiratory syndrome coronavirus 2 (SARS-CoV-2) has infected 0.776 billion individuals and caused 7.1 million deaths [11].

The SARS-CoV-2 virus is classified within the Coronaviridae family and belongs to the β-coronavirus genus [12]. Other members of this genus, including severe acute respiratory syndrome coronavirus (SARS-CoV), Middle East respiratory syndrome coronavirus (MERS-CoV), human coronavirus OC43 (HCoV-OC43), and human coronavirus HKU1 (HCoV-HKU1), also exhibit a propensity for human infection [[13], [14], [15]]. The genome of SARS-CoV-2 consists of a single-stranded positive-sense RNA approximately 29.8 kilobases (kb) in size, comprising 14 Open Reading Frames (ORFs). This genome encodes 27 proteins, both structural and non-structural. Two extensive polyproteins, pp1a and pp1ab, encoded by ORF-1a and ORF-1ab at the 5′-end of the genome, are responsible for synthesizing 15 non-structural proteins (nsp1–10 and nsp12–16). Key non-structural proteins include nsp3, a multifunctional protein containing the PL-pro domain; nsp5, a 3CL chymotrypsin-like protease; nsp9, a helicase likely involved in viral replication; nsp12, the RNA-dependent RNA polymerase (RdRp); and nsp13, a helicase enzyme. At the 3′ end of the genome, genetic information directs the synthesis of four structural proteins—spike surface glycoprotein (S), envelope protein (E), membrane protein (M), and nucleocapsid protein (N)—and eight auxiliary proteins, including 3a, 3b, p6, 7a, 7b, 8b, 9b, and orf14 [16]. Due to the high degree of diversity and rapid mutation, structural proteins, including spike (S) proteins, are considered less favorable targets for drug design. The ideal target for viral suppression is thought to be non-structural proteins like RdRp, such as nsp12, which are conserved. RdRp is essential for RNA genome replication and transcription, playing a major role in the viral life cycle. Furthermore, RdRp lacks a homolog in mammalian cells, and inhibiting it is not predicted to cause target-related side effects, making it a viable target for drug discovery and development [17]. Due to features such as exceptionally well-preserved genomic sequences, distinctive functions, and peculiar active sites, RdRp was selected as the target protein in this investigation [18].

Current efforts to combat the COVID-19 pandemic are focused on two primary strategies: the development of vaccinations for preventive measures and antiviral medications for therapeutic intervention. According to WHO, approximately 5.47 billion doses of various vaccines have been administered globally [19], each with varying risk-benefit profiles. However, the deployment of different vaccines has been associated with reports of serious adverse events [20,21]. Further research in this domain is essential to refine existing vaccines, enhance their efficacy, and mitigate potential side effects. In the pursuit of developing antiviral drugs targeting RdRp, a variety of natural compounds including alkaloids [22,23], flavonoids [24,25], triterpenes [26], and polycyclic aromatic compounds [27] have been assessed using *in silico* models. Additionally, FDA-approved drugs such as remdesivir [28], molnupiravir [29], galidesivir [30], ribavirin [31], favipiravir [32], and others [33] have been repurposed and repositioned to treat the COVID-19 patients by inhibiting the catalytic activity of RdRp.

China approved the antiviral drug favipiravir (6-fluoro-3-hydroxy-2-pyrazinecarboxamide) as a treatment option for the SARS-CoV-2 virus on February 15, 2020 [32]. Favipiravir functions as a prodrug that remains inactive against viral RNA polymerase until it undergoes intracellular phosphoribosylation to form favipiravir-ribofuranosyl-50-triphosphate (favipiravir-RTP). Favipiravir-RTP then binds to the active site of RdRp, inhibiting RNA replication. It has been proposed that human hypoxanthine guanine phosphoribosyl-transferase (HGPRT) facilitates the activation of favipiravir. This active form of favipiravir specifically targets the catalytic domain of viral RdRp, thereby blocking its enzymatic function. Importantly, favipiravir exhibits non-toxicity towards mammalian cells and does not interfere with RNA or DNA synthesis within these cells [34]. Favipiravir has also been successfully used to treat patients with mild-to-moderate sickness. However, its efficacy is constrained in patients with COVID-19 and requires further improvement and in-depth study [35]. This study involved the design of new analogs of favipiravir through the substitution of the fluorine atom on the pyrazine ring with various heterocyclic and homocyclic moieties using the Suzuki Cross Coupling reaction [36]. Both rings possess significant pharmacological implications [37]. Consequently, the fluorine atom on the pyrazine ring was substituted with these moieties, and their effects on drug-like properties and binding affinity towards the RdRp of SARS-CoV-2 were evaluated using *in silico* models. Druggability of new analogs was explored using ADMETox, and binding affinity was studied using molecular docking. Molecular dynamics simulations (MDS) were performed to better understand mechanistic insights and validate the docking results.

## 2 Methodology

### 2.1 .ADME and toxicity prediction

The ADME characteristics of selected ligands (**L1-L7**) were predicted using the SwissADME service (www.swissadme.ch/index.php) [38], while their toxicity profiles were assessed using the ProTox-II online tool [39]. Physicochemical properties such as the number of rotatable bonds, H-bond acceptors, H-bond donors, total polar surface area (TPSA), and degree of saturation were analyzed. Additionally, lipophilicity (consensus Log Po/w), aqueous solubility (Log S (ESOL)), pharmacokinetic parameters (gastrointestinal absorption, blood-brain barrier permeability, P-glycoprotein substrate status, Log Kp, and potential inhibitors), and drug-likeness criteria (based on Lipinski's rule [40] and bioavailability scores) were evaluated. Medicinal chemistry aspects such as PAINS and Brenk alerts, as well as synthetic accessibility, were also considered. Toxicity assessment included hepatotoxicity, carcinogenicity, immunotoxicity, mutagenicity, cytotoxicity, and determination of median lethal dose (LD50).

### 2.2 Ligands and protein preparation

**Fig. 1:**
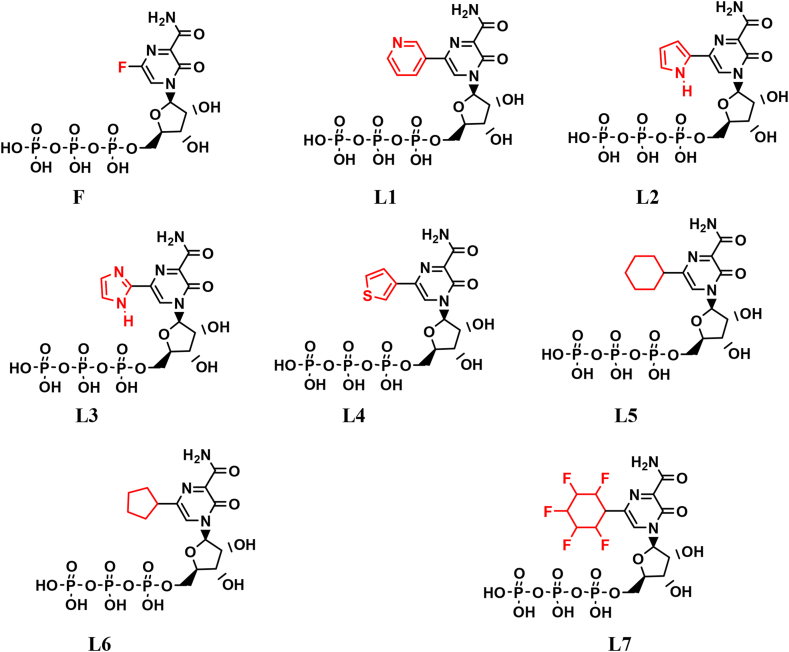
Biotransformed forms of favipiravir F and their new analogs (**L1-L7**).

The proposed novel inhibitors were drawn using ChemDraw (2019) software [41] and saved in. mol format (Fig. 1). These structures were subsequently converted to. PDB format using Open Babel [42]. Prior to docking, energy minimization was performed on these structures using the MMFF94 force field technique. Following this, the structures were converted to. PDBQT format using the Open Babel module in PyRx [43]. The crystal structure of RdRp (PDB ID: 7C2K) from SARS-CoV-2 [44] at a resolution of 2.93 Å was retrieved from the Protein Data Bank (www.rcsb.org). The RdRp structure was prepared by removing non-essential water molecules, adding all missing hydrogen atoms, and correcting charges. Energy minimization of RdRp was carried out using the Amber99 force field within the Molecular Operating Environment (MOE) software [45]. An active site identification tool integrated into MOE was utilized for the localization and characterization of active sites within RdRp.

### 2.3 Molecular docking analysis

The designated inhibitors (**L1-L7**) and favipiravir F were docked against RdRp using AutoDock Vina [46,47] integrated into PyRx software [43]. During the docking processes, the ligands were kept flexible, while the protein structure was treated as fixed using the Lamarckian Genetic Algorithm (LGA). The exhaustiveness value and number of binding modes were set to 8. The docking site on RdRp was defined by establishing a grid box with dimensions of 40 Å × 40 Å × 40 Å for the x, y, and z axes, respectively, with a grid spacing of 0.375 Å. Thirty runs with AutoDock Vina were performed for each ligand structure, and in each case, the best pose was saved based on the lowest docked energy. The interactions between RdRp and ligand conformations, including hydrogen bonds and bond lengths, were analyzed using Discovery Studio [48]. Similarly, the inhibition constant (Ki value) was also calculated using the equation Ki = е^-deltaG/RT^, where deltaG represents the binding free energy, R is the gas constant (1.987cal k^−1^ mol^−1^), and T is the temperature (298.1 K) [49].

### 2.4 Molecular dynamics simulation analysis

To better understand mechanistic insights and validate the docking results, structures of protein-ligand complexes involving the top-ranked three ligands (**L4**, **L6**, and **L7**) and parent drug favipiravir F were subjected to 200 ns MDS. The RdRp protein is large and requires more time for binding kinetics, conformational changes, and achieving equilibrium. Therefore, a 200 ns MDS was employed. The CHARMM 36 force field embedded in NAMD software was used for MDS. It was chosen for its high accuracy, versatility, and extensive parameterization specifically designed for biomolecular simulations [50]. VMD software was employed to prepare complex structures for MDS [51]. The complexes were equilibrated using the CHARMM GUI web server and subsequently run for 200 ns on a cluster computer [52]. For equilibration, all protein-ligand complex systems were solvated in the TIP3P water model. Simulation systems were kept electrically neutral (pH 7) by adding a 0.154 M NaCl solution at 310 K temperature and 1 atm pressure [53]. Trajectories and cluster analyses were performed using VMD and Chimera software from UCSF [54].

### 2.5 MM/GBSA analysis

The molecular mechanics generalized born surface area (MM-GBSA) module of prime was employed to compute the binding free energy (ΔG_bind_) of docked complexes [55]. The binding free energy was estimated using the VSGB solvent model [56], an OPLS 2005 force field [57], and the rotamer search algorithm. The total binding free energy was calculated using the following equation:

dG_bind_ = G_complex_ - (G_protein_ + G_ligand_)

Here, dG_bind_ = binding free energy, dG_complex_ = free energy of the complex, dG_protein_ = free energy of the target protein, and dG_ligand_ = free energy of the ligand.

## 3 Results and discussion

### 3.1 ADME and toxicity prediction

The drug discovery process depends heavily on ADME (adsorption, distribution, metabolism and excretion) and toxicity prediction, which provide information that helps choose safer and more potent drug candidates while reducing development costs and time. The toxicity results in Table 1 indicate that all new analogs (**L1-L7**) were found to be inactive against carcinogenicity, immunotoxicity, mutagenicity, and cytotoxicity, which is noteworthy. However, ligands (**L1-L4**) exhibited slight activity in the liver, whereas drugs (**L5-L7**) showed no hepatotoxicity. The LD50, representing the median lethal dose causing death in 50 % of test subjects, ranged from 575 to 2000 mg kg^−1^ for all compounds, classifying them as toxic class 4 [39], [58]. These results indicate that all new analogs are predicted to be orally non-toxic. Due to their non-toxic nature, favipiravir **F** and the new analogs underwent screening for important parameters such as lipophilicity, water solubility, pharmacokinetics, drug-likeness, medicinal chemistry, and physicochemical properties.

**Table 1:** ADME and toxicity profile of the selected favipiravir's derivatives.

Properties	L1	L2	L3	L4	L5	L6	L7	F
Num. rotatable bonds	02	02	02	02	02	02	02	01
Num. H-bond acceptors	05	04	05	04	04	04	09	05
Num. H-bond donors	02	03	03	02	02	02	02	02
Fraction Csp3	0.00	0.00	0.00	0.00	0.55	0.50	0.55	0.00
TPSA (A^2^)	101.99	104.89	117.78	117.34	89.10	89.10	89.10	89.10
Log P_o/w_	0.17	0.04	−0.40	0.86	1.23	0.89	1.52	−0.16
Log S	−1.67	−1.52	−1.12	−2.16	−2.44	−2.03	−2.76	−1.13
GI absorption	High	High	High	High	High	High	High	High
BBB permeant	No	No	No	No	No	No	No	No
P-gb substrate	No	No	No	No	No	No	No	No
CYP1A2	Yes	No	No	No	No	No	No	No
CYP2C19	No	No	No	No	No	No	No	No
CYP2C9	No	No	No	No	No	No	No	No
CYP2D6	No	No	No	No	No	No	No	No
Log K_p_ (cms^−1^)	−7.54	−7.54	−8.01	−7.04	−6.43	−6.73	−7.18	−7.29
Lipinski's rules	Yes	Yes	Yes	Yes	Yes	Yes	Yes	Yes
BS	0.55	0.55	0.55	0.55	0.55	0.55	0.55	0.55
PAINS (alert)	0	0	0	0	0	0	0	0
Brenk (alert)	0	0	0	0	0	0	0	0
Synthetic accessibility	2.44	2.59	2.45	2.54	2.65	2.54	4.15	2.03
Hepatotoxicity	Slightly active	Slightly active	Slightly active	Slightly active	Inactive	Inactive	Inactive	Inactive
Carcino	Inactive	Inactive	Inactive	Inactive	Inactive	Inactive	Inactive	Inactive
Immuno	Inactive	Inactive	Inactive	Inactive	Inactive	Inactive	Inactive	Inactive
Mutagenicity	Inactive	Inactive	Inactive	Inactive	Inactive	Inactive	Inactive	Inactive
Cytotoxicity	Inactive	Inactive	Inactive	Inactive	Inactive	Inactive	Inactive	Inactive
LD_50_ (mg kg^−1^)	575	1000	2000	800	1356	1356	2000	

**Fig. 2:**
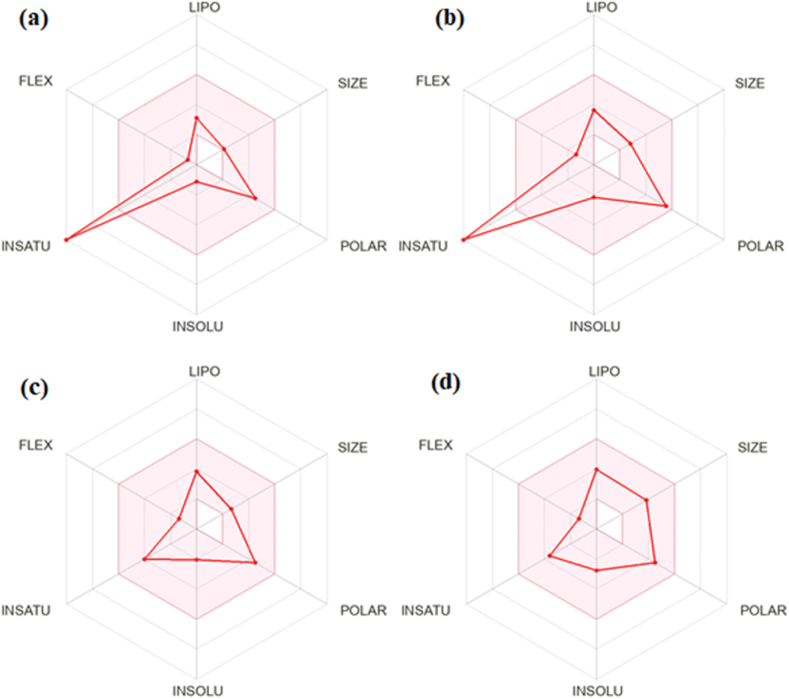
Bioavailability radar of (a) favipiravir F, (b) L4, (c) L6, and (d) L7. The pink area represents the optimal range for each property (solubility: log S not higher than 6, lipophilicity: XLOGP3 between −0.7 and + 5.0, saturation: fraction of carbons in the sp3 hybridization not less than 0.25, size: TPSA between 20 and 130Å^2^, and flexibility: no more than 9 rotatable bonds.

The bioavailability radar swiftly assesses drug-likeness based on six physicochemical properties: size, lipophilicity, polarity, solubility, flexibility, and saturation, each with an optimal range represented by a pink area [59,60]. A molecule is considered drug-like if all six parameters fall within this pink area. The radar indicated high unsaturation for favipiravir and its derivatives, except for ligands **L6** and **L7**. High unsaturation typically suggests poor oral bioavailability, yet favipiravir, despite its high unsaturation, is still administered orally as a medicine. Similarly, high unsaturation may not necessarily compromise the oral bioavailability of all derivatives, suggesting they could still be viable drug candidates. Interestingly, all six physicochemical properties of ligands **L6** and **L7** fell within the pink area, predicting their oral bioavailability and drug-likeness (Fig. 2). The optimal oral bioavailability of ligands **L6** and **L7** is attributed to the reduction in unsaturation achieved by substituting fluorine with cyclopentyl and hexafluorocyclohexyl moieties on their pyrazine rings, respectively.

The pharmacokinetics analysis revealed that favipiravir and its derivatives exhibit high gastrointestinal (GI) absorption, do not penetrate the blood-brain barrier (BBB), and do not interact with glycoprotein (P-gp). It was also predicted that these compounds generally do not interact with cytochromes, except for **L1**. Pharmacokinetic predictions indicate maximum bioavailability for all compounds, a critical requirement for effective drugs. Furthermore, each molecule displayed a 55 % bioavailability score, with no pain points or breaches of Lipinski's guidelines. Additionally, the evaluation of molecular structure complexity based on synthetic accessibility yielded scores ranging from 2.44 to 4.15 (Table 1). The synthetic accessibility score ranges from 1 (very easy) to 10 (very difficult). These findings indicate that the compounds examined can be synthesized using relatively simple routes. Based on pharmacokinetics and toxicity screening, all ligands are viable lead candidates. However, certain derivatives, such as **L6** and **L7**, show promising profiles and may perform better than favipiravir.

### 3.2 Interactions analysis

**Fig. 3:**
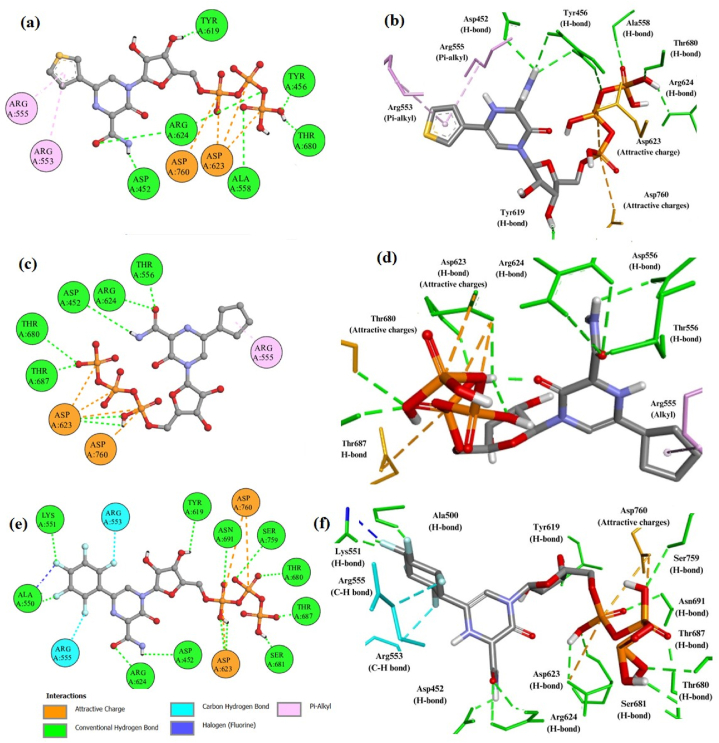
Two-dimensional and three-dimensional representations summarize the interaction analysis results for the (a, b) RdRp-L4 complex; (c, d) RdRp-L6 complex; and (e, f) RdRp-L7 complex, respectively. Pi-alkyl interactions are depicted as pink dashed lines, halogen (fluorine) interactions as blue dashed lines, attractive charges as orange dashed lines, carbon-hydrogen bonds as aqua dashed lines, and conventional hydrogen bonds as green dashed lines.

Based on the optimal ADMETox profile, all ligands underwent screening for docking studies to assess their potential affinity with the catalytic residues of RdRp. Docking results revealed that favipiravir (**F**) and the designated compounds (**L1-L7**) exhibited notable interactions with RdRp. Notably, **L7**, **L6**, and **L4** demonstrated the most favorable binding energies of −10.4 kcal mol^−1^, -9.6 kcal mol^−1^, and -9.4 kcal mol^−1^, respectively, ranking them first, second, and third (Table 2). Fig. 3 illustrates the interactions of these top-ranked ligands with RdRp. As a reference inhibitor, the nucleoside analog favipiravir displayed a binding energy of −8.0 kcal mol^−1^ (Table 2), which was comparatively lower among all screened compounds (Fig. S5). The Ki value, or inhibition constant, was calculated as the dissociation constant of the docked protein-ligand complex. A smaller Ki value indicates a lower probability of dissociation and therefore higher inhibition. Our top-ranked ligands L7, L6, and L4 showed Ki values of 0.0231 μM, 0.0823 μM, and 0.124 μM, respectively (Table 2). This clearly indicates that these top ligands have greater inhibition potential compared to all screened ligands and the parent drug favipiravir (F). The details regarding the interactions between the ligands and catalytic residues of RdRp are given below:

**Table 2:** Interaction details of favipiravir F and their new analogs (**L1-L7**) with RdRp.

Ligands	Binding energy (kcal mol^−1^)	Inhibition constant Ki (μM)	Nature of interactions	Catalytic residues
F	−8.0	1.34	H-bond	Met542, Ala558, Asp623, Arg624, Ser681, Ser682, Thr687
			C-H bond	Arg553, Arg555, Lys676, Ser681
			Pi-cation	Arg553
			Pi-alkyl	Arg555
L1	−9.1	0.212	H-bond	Cys622, Arg553, Asp452, Asp618
			C-H bond	Arg553
			Attractive charge	Asp671, Asp618, Glu811
			Pi-anion	Asp623
L2	−9.1	0.212	H-bond	Asp618, Tyr619, Asp623, Arg624, Thr680, Ser681, Asp760
			Pi-sigma	Lys621
			Sulfur-x	Met542
			Attractive charge	Asp623
L3	−8.7	0.443	H-bond	Arg553, Thr556, Asp623, Thr680, Ser681, Ser759
			Pi-alkyl	Ala558, Val557
			Pi-anion	Asp623
			Pi-sulfur	Met542
			Attractive charge	Asp452, Asp623
L4	−9.4	0.124	H-bond	Asp452, Tyr456, Ala558, Tyr619, Thr680, Arg624
			Pi-alkyl	Arg553, Arg555
			Attractive charge	Asp623, Asp760
L5	−9.1	0.212	H-bond	Asp452, Ala558, Tyr619, Arg624, Thr680, Ser682, Thr687, Asn691, Asp760,
			Pi-alkyl	Arg553
			Attractive charge	Asp623, Asp760
L6	−9.6	0.0823	H-bond	Asp452, Thr556, Asp623, Arg624, Thr680, Thr687
			Pi-alkyl	Arg553
			Attractive charge	Asp623, Asp760
L7	−10.4	0.0231	H-bond	Asp452, Ala550, Lys551, Tyr619 Asp623, Arg624, Thr680, Ser681, Thr687, Asn691, Ser759
			Fluorine	Ala550
			C-H bond	Arg553, Arg555
			Attractive charge	Asp623, Asp760

Ligand **L1** formed a stable protein-ligand complex by establishing 13 physical interactions, resulting in a binding energy of −9.1 kcal mol^−1^ (Table 2). Each catalytic residue, such as Cys622, Arg553, and Asp452, formed a single H-bond, respectively. Asp618 contributed two H-bonds. Arg553 also participated in a C-H interaction, and a pi-anion interaction was observed between the pyrazine moiety of **L1** and catalytic residue Asp623. Additionally, residues Asp671, Asp618, and Glu811 formed three, two, and one physical interactions (attractive charges) with phosphorus atoms of **L1**, respectively (Fig. S1).

Similarly, ligand **L2** bound stably to the active site of RdRp through 11 physical interactions, resulting in a binding energy of −9.1 kcal mol^−1^ (Table 2). Amino acids Asp618, Tyr619, Asp623, Arg624, Thr680, Ser681, and Asp760 each formed a single H-bond with **L2**. Asp623 contributed to two physical interactions (attractive charges) with phosphorus atoms of **L2**. Met542 formed a sulfur-x force interaction with **L2**, while Lys621 engaged in a pi-sigma interaction with the pyrrole moiety of ligand **L2** (Fig. S2).

Ligand **L3** formed a stable complex with RdRp, resulting in a binding energy of −8.7 kcal mol^−1^ (Table 2). Each catalytic residue, such as Arg553, Thr556, Thr680, Ser681, and Ser759, formed a single H-bond interaction. Asp623 contributed to two H-bond interactions. Additionally, amino acids Ala558 and Val557 each engaged in a single pi-alkyl interaction with the imidazole moiety of **L3**. Met542 formed a pi-sulfur interaction with the imidazole moiety of **L3**. Asp452 and Asp623 were involved in physical interactions (attractive charges) with phosphorus atoms of **L3**, respectively. Asp623 also participated in a pi-anion interaction with the imidazole moiety of **L3** (Fig. S3).

Similarly, ligand **L4** formed 13 physical interactions with the catalytic residues of RdRp, resulting in a binding energy of −9.4 kcal mol^−1^ (Table 2). Residues Asp452, Tyr456, Ala558, Tyr619, and Thr680 each formed a single H-bond, while Arg624 participated in two H-bond interactions. Additionally, residues Arg553 and Arg555 engaged in a single pi-alkyl interaction with the thiophene moiety of **L4**. Asp623 and Asp760 were involved in physical interactions (attractive charges) with phosphorus atoms of **L4**, respectively (Fig. 3a and b).

Similarly, ligand **L5** formed a stable protein-ligand complex by establishing 15 physical interactions, resulting in a binding energy of −9.1 kcal mol^−1^ (Table 2). Each catalytic residue—Asp452, Ala558, Tyr619, Arg624, Ser682, Thr687, Asn691, and Asp760—formed a single H-bond interaction, while Thr680 contributed to two H-bonds. Arg553 participated in a pi-alkyl interaction with the cyclohexyl ring of **L5**. Additionally, Asp623 and Asp760 were involved in three and one physical interactions (attractive charges) with phosphorus atoms of **L5**, respectively (Fig. S4).

Similarly, **L6** formed 12 non-covalent interactions with various catalytic residues of RdRp, resulting in a binding energy of −9.6 kcal mol^−1^ (Table 2). Amino acids such as Asp452, Thr556, Arg624, Thr680, and Thr687 each established a single H-bond, while Asp623 contributed to 2 H-bond interactions. Arg553 engaged in a pi-alkyl interaction with the cyclopentyl ring of **L6**. Additionally, Asp623 and Asp760 were involved in three and one physical interactions (attractive charges) with phosphorus atoms of **L6**, respectively (Fig. 3c and d).

Similarly, ligand **L7** formed a stable complex with RdRp, resulting in a more negative binding energy of −10.4 kcal mol^−1^ (Table 2). A total of 18 interactions of various types contributed to the high affinity of **L7** for RdRp. Each residue—such as Asp452, Ala550, Lys551, Tyr619, Arg624, Thr680, Ser681, Thr687, Asn691, and Ser759—formed a single H-bond with ligand **L7**. Additionally, Asp623 formed 2 H-bond interactions. Furthermore, residues Asp623 and Asp760 of RdRp were involved in one and two physical interactions (attractive charges) with two phosphorus atoms of **L7**, respectively. Catalytic residues Arg553 and Arg555 of RdRp established a single C-H interaction each. Ala550 also participated in a fluorine interaction with **L7** (Fig. 3e and f).

The docking results clearly demonstrate that all new analogs exhibit more negative binding energies and low inhibition constants compared to the parent drug favipiravir F (Table 2). These enhanced negative binding energies and low inhibition contants are primarily attributed to the incorporation of heterocyclic moieties such as pyrrole, imidazole, and thiophene in **L2**, **L3**, and **L4**, respectively, as well as homocyclic moieties including cyclohexyl, cyclopentyl, and hexafluorocyclohexyl in **L5**, **L6**, and **L7**, respectively. Furthermore, when compared against the triphosphate forms of galidesivir (−8.9 kcal mol^−1^), ribavirin (−9.8 kcal mol^−1^), and remdesivir (−9.2 kcal mol^−1^) [61], the top-ranked ligands **L4**, **L6**, and **L7** exhibit more negative binding energies than galidesivir, remdesivir, and in all cases except ribavirin, whose binding affinity surpasses that of **L4** and **L6**. Based on its binding energy, ligand **L7** has emerged as a potential inhibitor against the RdRp of SARS-CoV-2.

RdRp is structured into three domains: finger, palm, and thumb. The active site crucial for RdRp function resides within the palm domain, encompassing conserved polymerase motifs A to G [62]. Access of RNA templates to this active site, defined by motifs A (residues 611 to 626) and C (residues 753 to 767), occurs via a groove clamped by motifs F and G. The thumb subdomain and motif E provide structural support for the primer strand. Exiting the active site, the product-template hybrid follows the RNA exit tunnel situated anteriorly to the polymerase [63].

Residues Arg553 and Arg555 within motif F play a crucial role in forming the NTP entry channel. These residues interact with ligands (**L1, L3, L4, L5, L6**, and **L7**) in our study. Notably, the thiophene moiety of **L4** engages in pi-alkyl interactions with Arg553 and Arg555. Similarly, the cyclopentyl ring of **L6** forms a pi-alkyl interaction specifically with Arg553, while the hexafluorocyclohexyl ring of **L7** interacts through C-H bonds with both Arg553 and Arg555.

Our results highlight that the heterocyclic moiety (thiophene) of **L4**, and homocyclic moieties such as cyclopentyl and hexafluorocyclohexyl of **L6** and **L7**, respectively, are responsible for potentially inhibiting RdRp activity by obstructing the NTP entry channel. Additionally, residues Asp760 and Asp761 from motif C, pivotal for RdRp catalytic activity, engage with identified inhibitors [62,64,65]. Our investigation indicates that ligands (**L2, L4, L5, L6, and L7**) prominently interact with Asp760 through H-bonds and physical interactions (attractive charges), potentially impairing RdRp function.

### 3.3 Molecular dynamic simulation analysis

The complexes of ligands (**L4, L6**, and **L7**) and favipiravir **F** identified for their optimal ADMET profiles and docking scores, underwent a 200 ns MDS to elucidate their interaction patterns with the catalytic residues within the active site of RdRp. The MDS data, including root mean square deviation (RMSD), root mean square fluctuation (RMSF), protein-ligand contacts, 2D summary of protein-ligand interactions, and ligand property trajectories, are discussed in detail for each complex as follows.

#### 3.3.1 RdRp-L4 complex

**Fig. 4:**
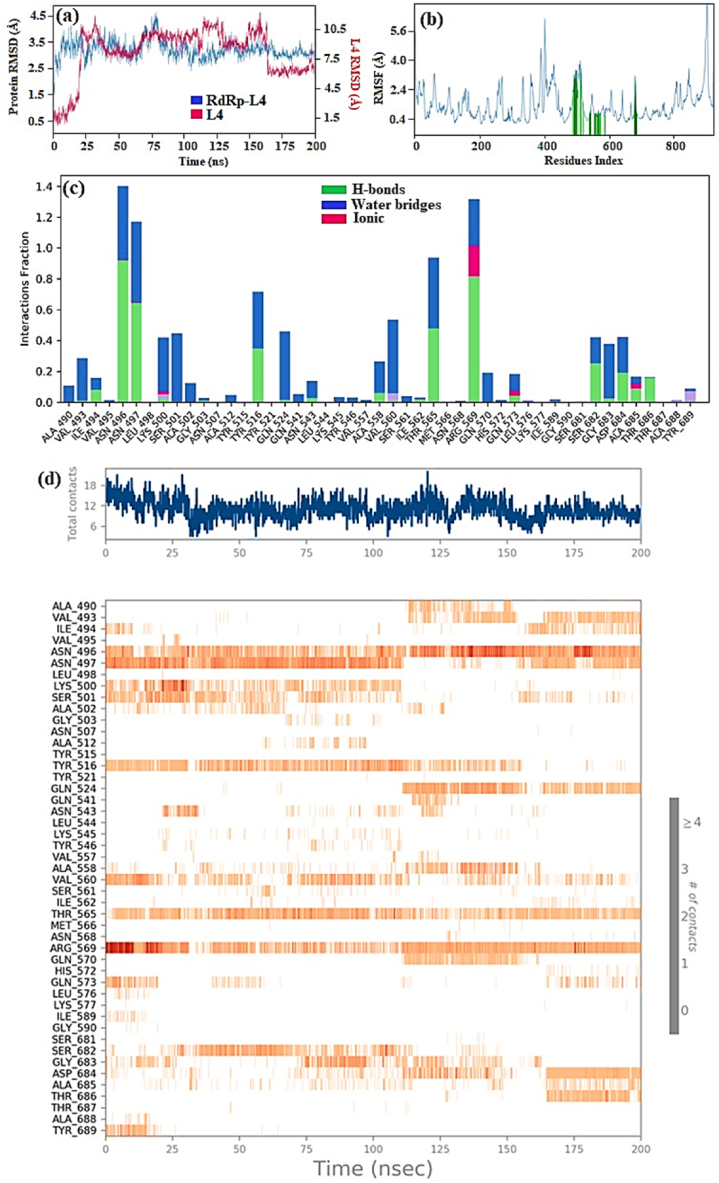
(a) Plot depicting RdRp and L4 RMSD values in RdRp-L4 complex over time, with RdRp RMSD on the left Y-axis and L4 RMSD on the right Y-axis; (b) RMSF of RdRp amino acid residues in RdRp-L4 complex; (c) The protein-ligand contact diagram of RdRp-L4 complex. The horizontal axis denotes residues involved in interactions with ligands, distinguished by color indicators representing the type of interaction; and (d) RdRp-L4 Interactions depicted in each trajectory frame involve active site amino acids, where absence of interactions is represented in white and stronger interactions are indicated by darker colors. All data were collected from a 200 ns MDS.

During the 200 ns simulation period of the RdRp-L4 complex, the RMSD analysis revealed that the RdRp initially showed a transient peak, reaching 4 Å, and even reaching 4.5 Å at 75 ns. This initial increase in RMSD suggests an early adjustment or perturbation in the protein's structure, possibly in response to simulation conditions or conformational changes induced by the binding environment. Subsequently, the RMSD curve stabilized, fluctuating between 3 Å and 3.5 Å, particularly notable from 75 ns to 200 ns, with an average RMSD of 3 Å (Fig. 4a). This indicates that the RMSD curve achieved significant equilibration following the initial perturbation and maintained stability with an average RMSD of 3 Å for the remainder of the simulation period.

Similarly, the RdRp-F complex displayed an average RMSD of 2.7 Å during the initial 60 ns, with minor fluctuations observed at 10 ns. However, the RMSD curve started to increase and peaked at 4.5 Å by 130 ns. Thereafter, it stabilized with an average RMSD of 4.2 Å, exhibiting occasional spikes for the remainder of the simulation period (Fig. S6).

Comparing the RMSD curves of the RdRp-L4 complex with the RdRp-F complex reveals the following insights: During the initial 75 ns, the RMSD curve of the RdRp-L4 complex fluctuates between 4 Å and 4.5 Å, indicating relatively lower stability compared to the RdRp-F complex, which maintains stability with an average RMSD of 2.7 Å over the initial 60 ns. Subsequently, from 75 ns to 200 ns, the RMSD curve of the RdRp-L4 complex stabilizes with an average RMSD of 3 Å, demonstrating its sustained stability. In contrast, the RMSD curve of the RdRp-F complex shows a significant increase up to 4.5 Å by 130 ns, followed by stabilization with an average RMSD of 4.2 Å for the remainder of the simulation period, indicating comparatively lower stability. Overall, the RdRp-L4 complex exhibits greater stability over a longer simulation period (75 ns–200 ns) compared to the RdRp-F complex (initial 60 ns).

Initially, the ligand **L4** displayed a modest RMSD value of 0.5 Å, gradually increasing to 1.5 Å over the initial 25 ns. Subsequently, a discernible shift occurred leading to a sudden elevation in RMSD to 4 Å and 4.5 Å between the 25 and 30 ns. However, as the simulation progressed, the ligand reached a balanced and equilibrated state maintaining an average RMSD of 3.5 Å for the remainder of the simulation. This suggests a stabilization of the ligand within the binding pocket of the protein.

The RMSF graph (Fig. 4b) display that Initially, the RMSF value was 5 Å, indicating a relatively higher degree of residue mobility. Subsequently, there was a noticeable reduction in RMSF, which decreased to 0.5 Å and remained within this reduced range up to the 399th residue. This decrease suggests a stabilization in the majority of residues, reflecting a more constrained molecular structure. However, residue 400 deviated from this trend, exhibiting fluctuations peaking at 6.4 Å. This increase in RMSF for residue 400 suggests greater mobility or structural flexibility in this specific residue, possibly indicating a region of the protein with distinct dynamic behavior compared to other residues. The catalytic residues highlighted in green bars showed minor variations within the standard range of 0.8–3.2 Å, indicating the formation of a stable protein-ligand complex.

The mode of action of the RdRp-L4 complex was further elucidated through detailed analysis of protein-ligand interactions, which included assessment based on protein-ligand contact and interaction scores. These interactions encompassed H-bonds (depicted in green), ionic interactions (marked in red), and water bridges (illustrated in blue) (Fig. 4c). These interactions collectively contribute to the stabilization of the protein-ligand complex. Among these, hydrogen bonding plays a pivotal role in exerting significant influence, followed by water bridges, while ionic and hydrophobic interactions contribute to a lesser extent.

**Fig. 5:**
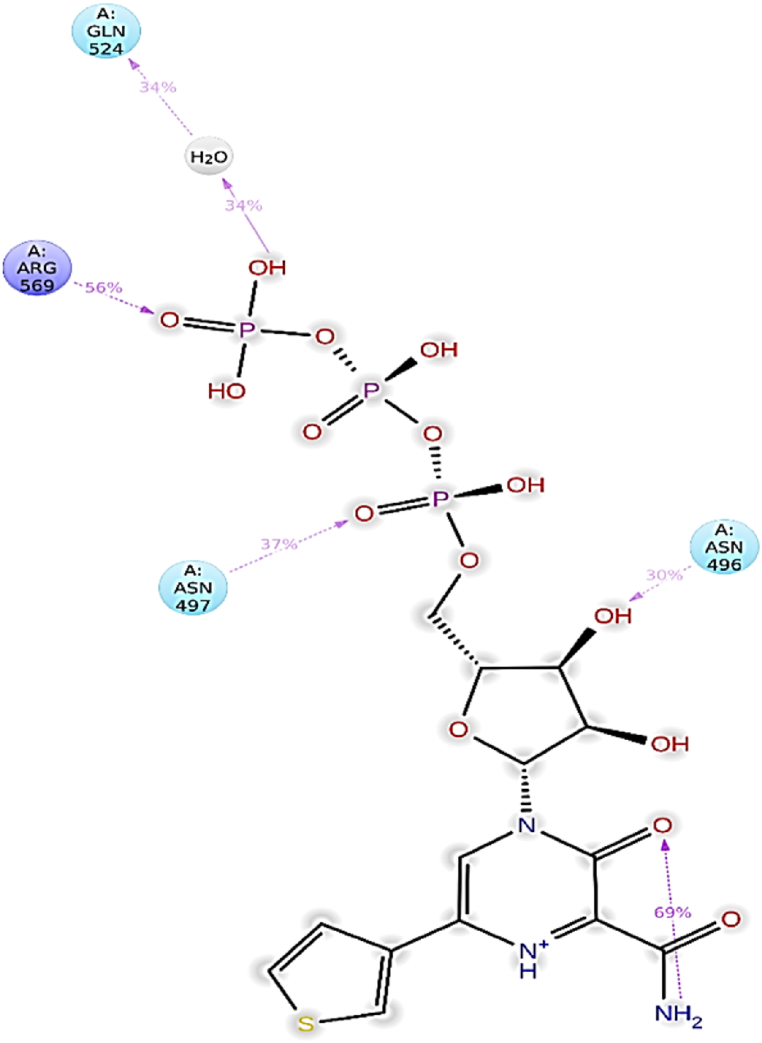
2D summary of percentage interactions between the effective residues of RdRp and the ligand L4 in RdRp-L4 complex across 200 ns MDS. Interactions occurring for more than 30 % of the simulation time are depicted.

The following residues formed water bridges with ligand **L4**: Ala490, Val493, Ile494, Val495, Asn496, Asn497, Lys500, Ser501, Ala502, Gly503, Ala512, Tyr516, Gln524, Gln541, Asn543, Lys545, Tyr546, Val557, Ala558, Val560, Ser561, Thr565, Arg569, Gln570, His572, Gln573, Ile589, Ser682, Gly683, and Asp684. Residues involved in hydrogen bonding include Ile494, Asn496, Asn497, Tyr526, Asn543, Ala558, Ile562, Thr565, Arg569, Gln574, Ser682, Asp684, Ala685, and Thr686. Ionic interactions involved the residues Lys500, Arg569, Gln574, and Ala685 (Fig. 4c). Catalytic residues such as Asn496, Asn497, and Arg569 maintained an interaction fraction (IF) greater than 0.1, indicating their sustained engagement in interactions throughout the 200 ns simulation period (a value of IF ≥ 0.1 signifies persistent involvement of a specific residue in interactions) (Fig. 4c). Fig. 5 presents a 2D summary of the protein-ligand contact analysis results detailing the interaction between RdRp and ligand **L4** over the 200 ns simulation period. Residues Asn496, Asn497, and Arg569 maintained H-bond interactions of 34 %, 37 %, and 56 %, respectively. Amino acid Gln524 exhibited 30 % involvement in water bridge interactions. The interaction profile revealed by docking analyses differs notably from that observed in RdRp-L4 contacts. Nevertheless, residue Ala558 consistently engages in H-bond interactions in both the docking analyses and protein-ligand contact assessments. These results indicate that various interactions, including hydrogen bonds, water bridges, and ionic interactions, contribute to the stability of the RdRp-L4 complex over the entire 200 ns simulation period.

The detailed analysis of RdRp-L4 contacts is also presented in Fig. 4d, featuring two panels for enhanced clarity. The upper panel illustrates total contacts ranging from 6 to 18, with an average of 12. In contrast, the lower panel provides a breakdown of individual residue interactions. Specifically, residues involved include Ala490, Val493, Ile494, Val495, Asn496, Asn497, Lys500, Ser501, Ala502, Gly503, Ala512, Tyr516, Gln524, Gln541, Asn543, Lys545, Tyr546, Val557, Ala558, Val560, Ser561, Thr565, Arg569, Gln570, His572, Gln573, Ile589, Ser682, Gly683, Asp684, Ile494, Asn496, Asn497, Tyr526, Asn543, Ala558, Ile562, Thr565, Arg569, Gln574, Ser682, Asp684, Ala685, Thr686, Lys500, Arg569, Gln574, and Ala685. These interactions persisting throughout the 200 ns simulation underscore their crucial role in stabilizing the RdRp-L4 complex and potentially inhibiting its function against SARS-CoV-2.

**Fig. 6:**
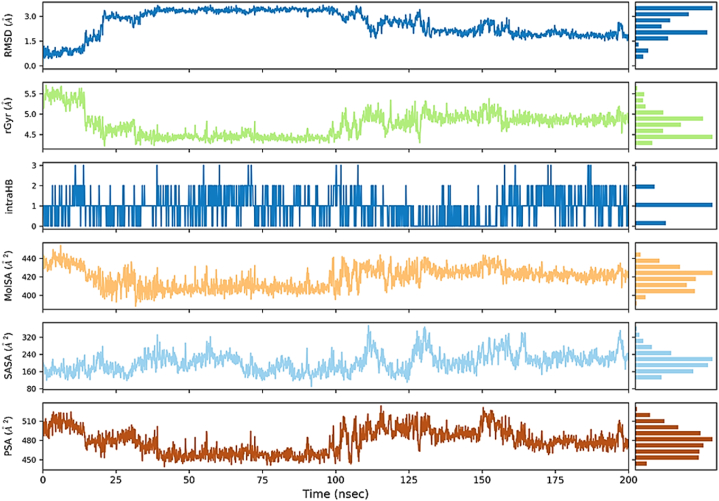
The ligand L4 property trajectoryies (RMSD, rGyr, intraHB, MolSA, SASA, and PSA) of RdRp-L4 complex through 200 ns MDS.

The stability analysis of ligand **L4** was conducted across six parameters during the 200 ns MDS, as depicted in Fig. 6. The RMSD values showed temporal fluctuations ranging from 0.5 Å to 3 Å, indicating dynamic structural variations over time. The radius of gyration (rGyr) exhibited a constrained range oscillating between 4.5 Å and 5 Å, suggesting minimal conformational changes throughout the simulation. Hydrogen bonding analysis revealed an average occurrence of 1–2 bonds, contributing to the ligand's molecular stability. Additionally, the molecular surface area (MolSA) demonstrated variability from 400 Å^2^ to 440 Å^2^, signifying alterations in the ligand's exposed surface area during the simulation. The solvent-accessible surface area (SASA) exhibited fluctuations between 160 Å^2^ and 200 Å^2^, with a consistent average value of 170 Å^2^ maintained throughout the entire simulation, indicative of interactions with the solvent and accessibility. Furthermore, the polar surface area (PSA) showed dynamic values ranging from 450 Å^2^ to 520 Å^2^, with an average value of 480 Å^2^ over the entire simulation period, emphasizing the ligand's polar characteristics.

#### 3.3.2 RdRp-L6 complex

**Fig. 7:**
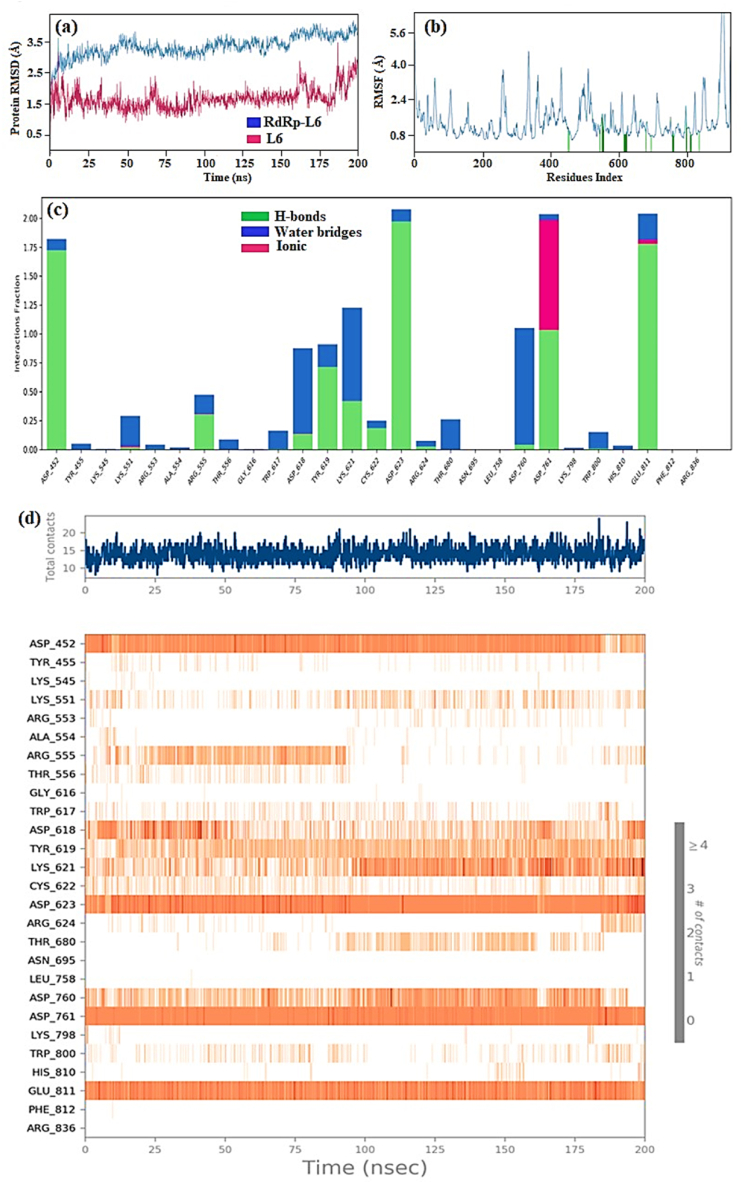
(a) Plot depicting RdRp and L6 RMSD values in RdRp-L6 complex over time, with RdRp RMSD on the left Y-axis and L6 RMSD on the right Y-axis; (b) RMSF of RdRp amino acid residues in RdRp-L6 complex; (c) The protein-ligand contact diagram of RdRp-L6 complex. The horizontal axis denotes residues involved in interactions with ligands, distinguished by color indicators representing the type of interaction; and (d) RdRp-L6 Interactions depicted in each trajectory frame involve active site amino acids, where absence of interactions is represented in white and stronger interactions are indicated by darker colors. All data were collected from a 200 ns MDS.)

The RMSD analysis initially showed that from 10 ns to 35 ns, the RdRp-L6 complex stabilized with an RMSD of 3 Å. Over the extended simulation period of 160 ns, it maintained stability within the range of 3 Å to 3.7 Å. However, a minor fluctuation at 170 ns caused an increase to 4 Å (Fig. 7a). Overall, the RdRp-L6 complex exhibited an average RMSD of 3.2 Å, indicating acceptable deviation and stability throughout the simulation period.

Comparing the RMSD curves of the RdRp-L6 complex with the RdRp-F complex provides further insights: The RdRp-L6 complex displays an average RMSD of 3.2 Å, highlighting its stability over the 200 ns simulation period. In contrast, the RdRp-F complex maintains stability with an average RMSD of 2.7 Å over the initial 60 ns. However, the RMSD curve of the RdRp-F complex shows a significant increase to 4.5 Å by 130 ns (Fig. S6), followed by stabilization with an average RMSD of 4.2 Å for the remaining simulation period, indicating comparatively lower stability. Overall, the RdRp-L6 complex demonstrates greater stability compared to the RdRp-F complex.

Similarly, for ligand **L6**, fluctuations were observed up to 25 ns, after which the curve stabilized within the range of 1.5 Å to 2 Å RMSD until 160 ns, providing clear evidence of stability for the protein-ligand complex (Fig. 7a). RMSD evaluation indicates that ligand **L6** induced conformational changes within the protein upon docking into the binding pocket and exhibited overall stability throughout the simulation.

The RMSF graph illustrates stable residue conformations of the protein throughout the simulation, with minor fluctuations within acceptable ranges. Specifically, residues 1 to 200 demonstrated predominantly below 3.2 Å fluctuations with notable stability. A triad of peaks emerged between residues 201 and 400, where fluctuations ranged from 3.5 Å to 4.5 Å. Similarly, residues 401 to 600 exhibited minor fluctuations, maintaining RMSF values within the range of 2 Å to 3.2 Å. This pattern persisted in the residue range of 600–800. In contrast, heightened fluctuations were observed between residues 900 and 1000, where RMSF values peaked at 6.4 Å. Modest fluctuations within the usual range of 0.8–1.6 Å were observed in catalytic residues indicated by green bars, suggesting the formation of a stable RdRp-L6 complex (Fig. 7b).

**Fig. 8:**
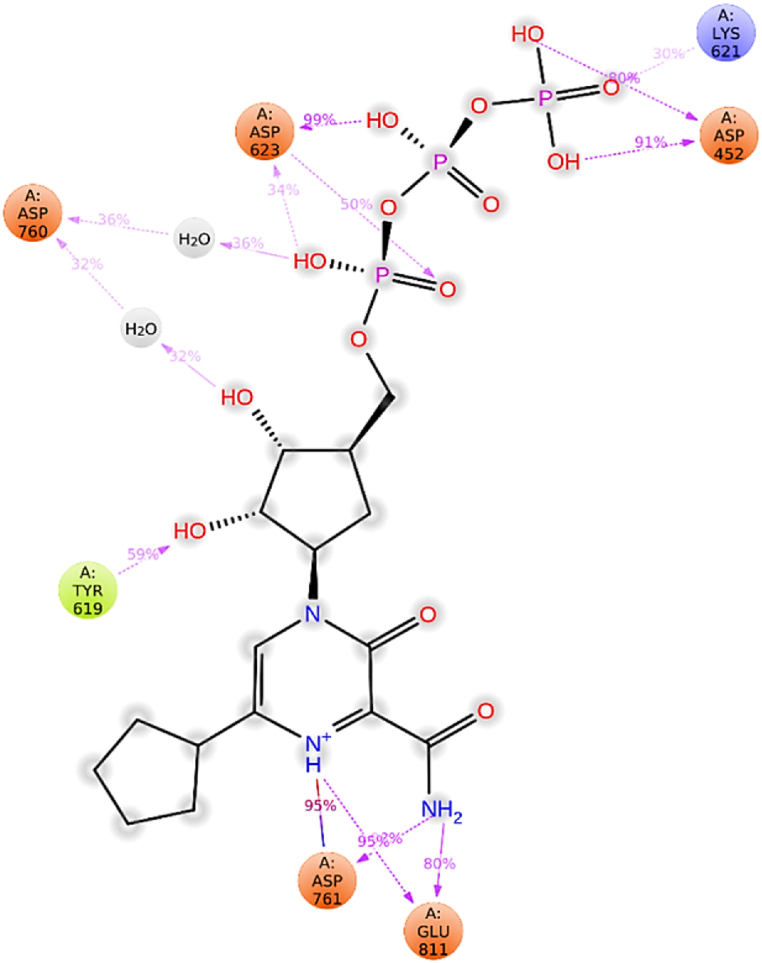
2D summary of percentage interactions between the effective residues of RdRp and the ligand L6 in RdRp-L6 complex across 200 ns MDS. Interactions occurring for more than 30 % of the simulation time are depicted.

Fig. 7c illustrates RdRp-L6 contacts, highlighting specific residues such as Asp452, Lys551, Arg555, Asp618, Tyr619, Cys622, Asp623, Arg624, Asp760, Asp761, and Glu811 involved in H-bonds. Additionally, Asp761 and Glu811 participated in ionic interactions. Water bridge interactions were observed for Asp452, Tyr455, Lys551, Arg553, Ala554, Trp617, Asp618, Tyr619, Lys621, Cys622, Asp623, Arg624, Thr680, Asp760, Lys798, Trp800, His810, Glu811, and Phe812. Residues Asp452, Tyr619, Asp623, Asp761, and Glu811 maintained interactions with an intensity (IF) greater than 0.1 throughout the 200 ns simulation. Fig. 8 provides a 2D summary of the protein-ligand contact analysis results depicting the interaction between RdRp and ligand **L6** throughout the 200 ns simulation period. The catalytic residue Asp452 demonstrated 91 % and 80 % involvement in H-bond interactions. Amino acids Tyr619 and Lys621 exhibited 59 % and 30 % participation in H-bond interactions, respectively. Asp623 was involved in 99 %, 50 %, and 34 % H-bond interactions. Water bridge interactions of 36 % and 32 % were observed for residue Asp760. Amino acid Asp761 displayed 95 % and 80 % engagement in H-bond interactions. Glu811 exhibited 95 % and 80 % participation in H-bond interactions.

It is noteworthy that amino acids such as Arg553 and Arg555 within motif F establish H-bond and water bridge interactions with ligand **L6**. Similarly, residue Asp760 in motif C participates in H-bond and water bridge interactions with Ligand **L6**, while Asp761 from motif C engages in an ionic interaction with **L6** and also maintains continuous contact throughout the simulation period. These interactions collectively indicate that **L6** inhibits RdRp by disrupting the catalytic activities associated with motifs F and C. These findings align consistently with the docking analysis results.

Fig. 7d provides a detailed depiction of RdRp-L6 interactions. The top panel presents an overview of total contacts, showing variability within the range of 10–15. In the bottom panel, it is evident that residues including Asp452, Lys551, Arg555, Asp618, Tyr619, Lys621, Asp623, Asp761, and Glu811 consistently maintained contact throughout the simulation period. The persistent occurrence of these interactions throughout the 200 ns simulation emphasizes their critical role in stabilizing the RdRp-L6 complex and potentially inhibiting its function against SARS-CoV-2.

**Fig. 9:**
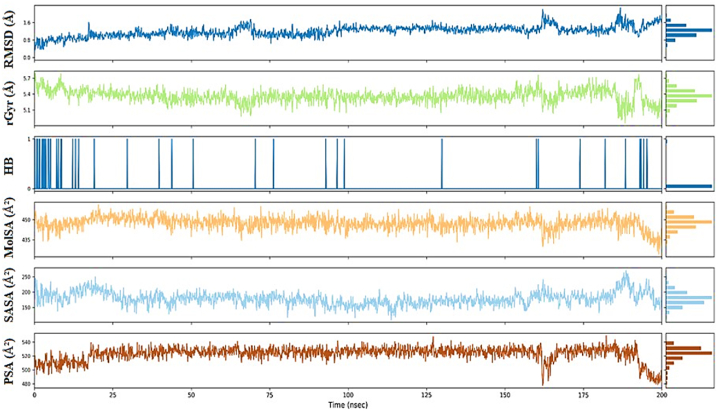
The ligand L6 property trajectoryies (RMSD, rGyr, intraHB, MolSA, SASA, and PSA) of RdRp-L6 complex through 200 ns MDS.

Fig. 9 illustrates the properties of ligand **L6**, revealing an RMSD value that remained stable at 1 Å throughout the entire 200 ns simulation period, indicating its structural stability. The radius of gyration (rGyr) was consistently observed at 5.4 Å, suggesting limited conformational changes over the course of the simulation. Hydrogen bonding analysis revealed an average occurrence of 1 bond, contributing to the ligand's molecular stability. The MolSA ranged between 4.4 Å^2^ and 4.5 Å^2^, indicating slight variations in the exposed surface of the ligand. The SASA fluctuated between 150 Å^2^ and 200 Å^2^, with an average value of 175 Å^2^ maintained throughout the simulation, reflecting solvent interactions and accessibility. The PSA showed a range of 500 Å^2^ to 520 Å^2^, with an average value consistently at 520 Å^2^ across the entire simulation period, underscoring the ligand's polar characteristics and interactions.

#### 3.3.3 RdRp-L7 complex

**Fig. 10:**
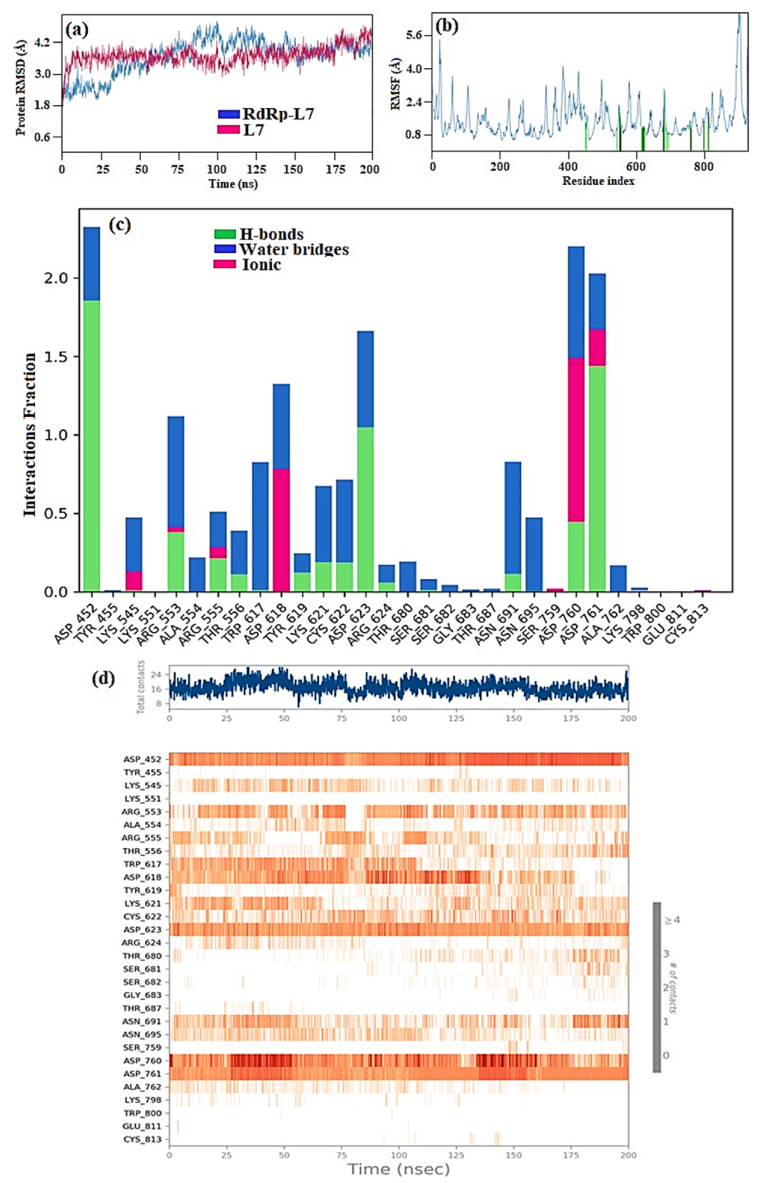
(a) Plot depicting RdRp and L7 RMSD values in RdRp-L7 complex over time, with RdRp RMSD on the left Y-axis and L6 RMSD on the right Y-axis; (b) RMSF of RdRp amino acid residues in RdRp-L7 complex; (c) The protein-ligand contact diagram of RdRp-L7 complex. The horizontal axis denotes residues involved in interactions with ligands, distinguished by color indicators representing the type of interaction; and (d) RdRp-L7 Interactions depicted in each trajectory frame involve active site amino acids, where absence of interactions is represented in white and stronger interactions are indicated by darker colors. All data were collected from a 200 ns MDS.

The RMSD analysis of the RdRp-L7 complex initially recorded a value of 2.4 Å at the simulation onset, maintaining this stability until 30 ns. Subsequently, the RMSD gradually increased to 4.8 Å by 100 ns and then decreased to 4.2 Å by 150 ns, remaining stable until 200 ns (Fig. 10a). Notably, between 100 ns and 200 ns, the complex maintained an average RMSD of 4.0 Å, indicating sustained stability throughout this simulation period.

A comparative analysis of the RMSD curves between the RdRp-L7 complex and the RdRp-F complex provides further insights: During the initial 30 ns, the RdRp-L7 complex exhibits an RMSD of 2.4 Å, indicating relatively higher stability compared to the RdRp-F complex, which maintains an average RMSD of 2.7 Å over the initial 60 ns. From 100 ns to 200 ns, the RMSD curve of the RdRp-L7 complex stabilizes at an average RMSD of 4.0 Å, demonstrating continued stability. In contrast, the RMSD curve of the RdRp-F complex shows a significant increase to 4.5 Å by 130 ns, followed by stabilization with an average RMSD of 4.2 Å (Fig. S6) for the remainder of the simulation period, indicating comparatively lower stability. Overall, the RdRp-L7 complex exhibits greater stability compared to the RdRp-F complex.

Similarly, the RMSD curve for the ligand **L7** started at 1.8 Å and rose to 3.6 Å by 10 ns. From 10 ns to 175 ns, the curve remained consistently at 3.6 Å, after which it increased to 4.2 Å and remained stable for the rest of the simulation, indicating the stability of the protein-ligand complex (Fig. 10a).

The RMSF graph showed values ranging from 0.8 Å to 2.4 Å for residues 1 to 200, with minor fluctuations touching 3.5 Å. From residue 201 to 400, the curve remained between 0.8 Å and 4 Å with a few fluctuations, similar to the pattern observed from residue 401 and above 800. Catalytic residues, marked by green bars, displayed small fluctuations within the normal range of 0.8–2.4 Å, indicating the formation of a stable protein-ligand complex (Fig. 10b).

**Fig. 11:**
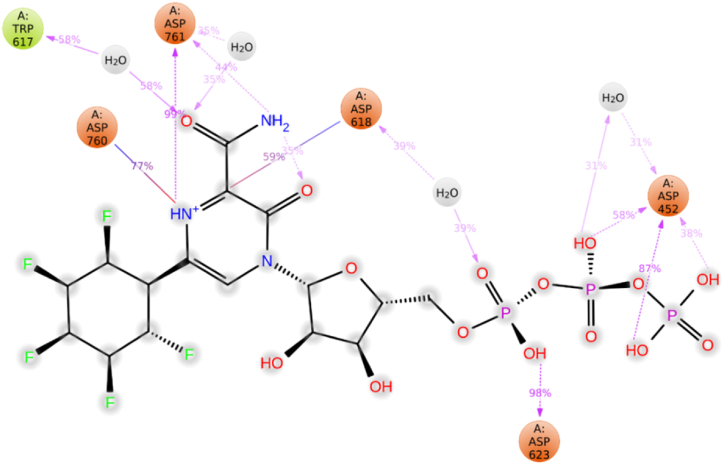
2D summary of percentage interactions between the effective residues of RdRp and the ligand L7 in RdRp-L7 complex across 200 ns MDS. Interactions occurring for more than 30 % of the simulation time are depicted.

The RdRp-L7 complex was further elucidated through an extensive analysis of protein-ligand interactions (Fig. 10c). Key residues such as Asp452, Arg553, Arg555, Thr556, Tyr619, Lys621, Cys622, Asp62, Arg624, Asn691, Asp760, and Asp761 were identified to engage in H-bond interactions. Additionally, amino acids including Lys545, Arg553, Arg555, Asp618, Ser759, Asp760, and Asp761 were observed to participate in ionic interactions. Water bridge interactions were documented involving residues such as Tyr455, Lys551, Arg553, Ala554, Arg555, Thr556, Gly616, Trp617, Asp618, Tyr619, Kys621, Cys622, Asp623, Arg624, Thr680, Asn695, Leu758, Asp760, Asp761, Lys798, Trp800, His810, Glu811, Phe812, and Arg836. Residues including Asp452, Asp618, Asp623, Asp760, and Asp761 demonstrated sustained interaction involvement throughout the 200 ns simulation period, with IF greater than 01. Fig. 11 presents a 2D summary of the protein-ligand contact analysis results for RdRp binding with ligand **L7** over the 200 ns simulation period. Amino acid Asp452 expressed 87 %, 58 %, and 38 % H-bond interactions along with 31 % of water bridge interactions. Residues Asp623 and Asp760 maintained 98 %, and 77 % H-bond interactions, respectively. Residue Asp618 exhibited 59 % and 39 % H-bond and water bridge interactions. Asp761 exhibited 99 % and 44 % H-bond and 35 % water bridge interactions. Trp617 displayed 58 % water bridge interaction.

Amino acids Arg553 and Arg555 within motif F, alongside residues Asp760 and Asp761 in motif C, exhibit significant interactions including H-bonds, water bridges, and ionic interactions with the ligand **L7**. Notably, Asp760 and Asp761 maintain continuous contact throughout the simulation period. These interactions suggest that L7 inhibits RdRp by disrupting catalytic activities linked to motifs F and C. These findings are consistent with the results obtained from docking analysis.

The intricate interactions between RdRp and ligand **L7** are thoroughly detailed in Fig. 10d. The upper panel provides an overview of total contacts, showing variability ranging from 8 to 24 contacts with an average of 16 interactions observed throughout the simulation. In the lower panel, specific residues—Asp452, Lys551, Arg555, Asp618, Tyr619, Lys621, Asp623, Asp761, and Glu811—were highlighted for their sustained contact duration during 200 ns simulations. Notably, interactions with residues such as Asp452, Asp623, Asp760, Tyr619, and Asp761 persistently maintained over the extended simulation period, affirming the long-term stability of the RdRp-L7 complex in simulated conditions. The persistent presence of these interactions throughout the 200 ns simulations highlights their critical role in maintaining the stability and potential inhibitory effect of the RdRp-L7 complex against SARS-CoV-2.

**Fig. 12:**
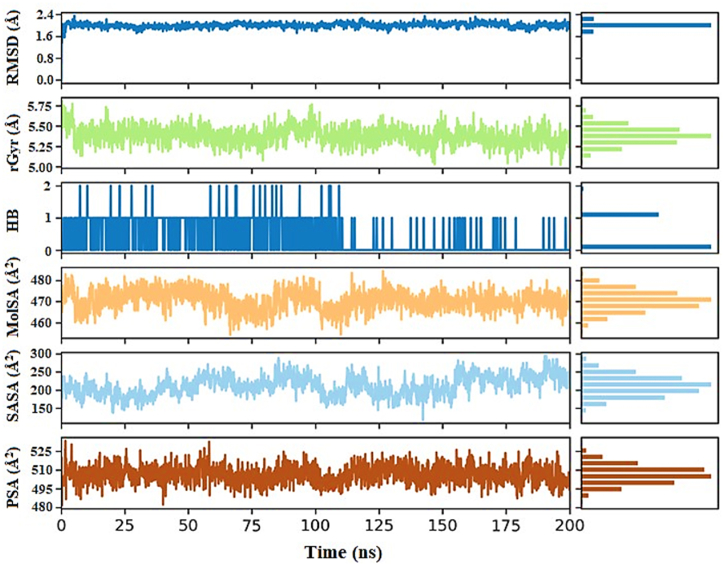
The ligand L7 property trajectoryies (RMSD, rGyr, intraHB, MolSA, SASA, and PSA) of RdRp-L7 complex through 200 ns MDS.

Fig. 12 provides a detailed depiction of the dynamic characteristics of the ligand **L7**. The RMSD value remained stable at 2.2 Å, indicating consistent structural behavior. The radius of gyration (rGyr) maintained a constant value of 5.50 Å, suggesting minimal conformational changes over time. Hydrogen bonding analysis indicated the presence of 1–2 bonds on average, which played a crucial role in maintaining the ligand's molecular stability. The MolSA exhibited slight oscillations within the narrow range of 4.7 Å^2^ to 4.8 Å^2^, indicating minor variations in the ligand's exposed surface area. The SASA fluctuated between 200 Å^2^ and 250 Å^2^, with an average value of 225 Å^2^ maintained throughout the simulation period, reflecting solvent interactions and accessibility. Meanwhile, the PSA showed a broader range oscillating between 495 Å^2^ and 510 Å^2^, yet the average value remained constant at 500 Å^2^ over the entire simulation period.

### 3.4 MM/GBSA calculations

The precision of binding energy calculated through docking techniques is often limited, making it challenging to distinguish between docked poses with similar binding orientations and affinities. To overcome this limitation, MM/GBSA has proven to be a reliable and accurate method for estimating the structural stability and binding energy of protein-ligand complexes. The total binding energy (G_bind_) comprises several components such as G_bind_Coulomb, G_bind_Packing, G_bind_H_bond_, G_bind_Lipo, and G_bind_vdW, which are governed by non-bonded interactions. Among these, G_bind_vdW, G_bind_Lipo, G_bind_Coulomb energies typically exert the greatest influence on the average binding energy across all interaction types.

In this study, the free binding energy of the top-ranked ligands was assessed using MM/GBSA. Ligand **L7** exhibited the highest binding energy of −66.233 kcal mol^−1^, followed by **L4** (−62.245 kcal mol^−1^), L6 (−54.221 kcal mol^−1^), and favipiravir (−51.744 kcal mol^−1^) (Table 3). These findings underscore the consistency between docking data and MM/GBSA estimates derived from MD simulation trajectories.

**Table 3:** Average MM/GBSA binding energy calculations of top ranked ligands (L7, L6 and L4) and favipiravir F.

**Energies (kcal mol**^**−**^**^1^)**	L7	L6	L4	F
**dG**_**bind**_	−66.233	−54.221	−62.245	−51.744
**dG**_**bind**_**Lipo**	−4.841	−30.671	−2.438	−36.9817
**dG**_**bind**_**vdW**	−42.406	−60.802	−42.117	−39.732
**dG**_**bind**_**Coulomb**	−72.367	−102.36	−68.161	−33.279
**dG**_**bind**_**H**_**bond**_	−8.402	−3.237	−5.412	−1.672
**dG**_**bind**_**Packing**	−0.1861	−1.933	−1.423	−0.372

## 4 Conclusions

In conclusion, ligands **L6** and **L7** demonstrate superior bioavailability and enhanced drug-likeness compared to the parent drug favipiravir (F). This improved oral bioavailability and druggability stem from reduced unsaturation achieved by substituting fluorine with cyclopentyl and hexafluorocyclohexyl moieties on their pyrazine rings, respectively. Moreover, these ligands can be synthesized via relatively simple routes, with synthetic accessibility scores of 2.54 and 4.15 for **L6** and **L7**, respectively, on a scale ranging from 1 (very easy) to 10 (very difficult). The designated analogs demonstrate strong inhibition potential, characterized by their low inhibition constants and strong binding affinity to the active site of RdRp compared to the parent drug favipiravir (F). These attributes, including enhanced negative binding energies, are primarily attributed to the incorporation of heterocyclic moieties such as pyrrole, imidazole, and thiophene in **L2**, **L3**, and **L4**, respectively, as well as homocyclic moieties such as cyclohexyl, cyclopentyl, and hexafluorocyclohexyl in **L5**, **L6**, and **L7**, respectively. Ligands **L4**, **L6**, and **L7** are ranked as the top three due to their highly negative binding energies of −9.4 kcal mol^−1^, -9.6 kcal mol^−1^, and -10.4 kcal mol^−1^, respectively. Our findings underscore that the thiophene moiety in **L4** and homocyclic moieties like cyclopentyl and hexafluorocyclohexyl in **L6** and **L7**, respectively, contribute significantly to inhibiting RdRp activity by obstructing the NTP entry channel. MD simulations validate the docking analyses of these top-ranked ligands, showing their relatively high stability over the simulation period compared to favipiravir. Key interactions such as H-bonds, pi-alkyl interactions, C-H bonds, attractive charges, water bridges, and ionic interactions are pivotal in binding these ligands to the active site of RdRp. This theoretical study suggests that ligands **L6** and **L7** could serve as lead candidates for inhibiting RdRp of SARS-CoV-2. Further experimental studies, such as Cell-Based SARS-CoV-2 RdRp Activity Assays, are recommended to validate these *in silico* findings.

## Funding

Researchers Supporting Project number (RSP2024R110), 10.13039/501100002383King Saud University, Riyadh, Saudi Arabia.

## CRediT authorship contribution statement

**Saira:** Methodology, Investigation, Formal analysis, Data curation. **Khalid Khan:** Writing – original draft, Project administration, Methodology, Formal analysis, Conceptualization. **Asad Khan:** Software, Methodology, Investigation, Formal analysis, Data curation. **Ateeq Khan:** Methodology, Investigation, Funding acquisition, Formal analysis, Conceptualization. **Tanzeel Shah:** Writing – original draft, Supervision, Formal analysis, Conceptualization. **Nasir Ahmad:** Supervision, Software, Methodology, Data curation, Conceptualization. **Haroon ur Rashid:** Writing – review & editing, Writing – original draft, Supervision, Investigation, Formal analysis, Conceptualization. **Muhammad Zahoor:** Writing – review & editing, Writing – original draft, Supervision, Project administration, Methodology, Conceptualization. **Riaz Ullah:** Writing – original draft, Project administration, Methodology, Conceptualization. **Ahmed Bari:** Methodology, Investigation, Funding acquisition, Formal analysis, Data curation, Conceptualization. **Muhammad Naveed Umar:** Methodology, Investigation, Funding acquisition, Formal analysis, Data curation, Conceptualization.

## Declaration of competing interest

The authors declare that they have no known competing financial interests or personal relationships that could have appeared to influence the work reported in this paper.
