# Supplementary material for: In silico evaluation of favipiravir-associated potential new drugs against polymerase enzyme of SARS-CoV-2
Source: Heliyon. 2024 Sep 26;10(19):e38479. doi: 10.1016/j.heliyon.2024.e38479 (PMC11467532; doi:10.1016/j.heliyon.2024.e38479)
Supplement: Multimedia component 1 [file mmc1.docx]

**Supplementary Materials**

Saira^1^, Khalid Khan^1*^, Asad Khan^1^, Ateeq Khan^1^, Tanzeel Shah^2^, Nasir Ahmad^1^, Haroon ur Rashid^3^, Muhammad Zahoor^4*^, Riaz Ullah^5^, Ahmed Bari^6^, Muhammad Naveed Umar^7^

1. Department of Chemistry, Islamia College Peshawar, Khyber Pakhtunkhwa, 25120, Pakistan; [sairaphd@icp.edu.pk](mailto:sairaphd@icp.edu.pk) , [drkhalidchem@yahoo.com](mailto:drkhalidchem@yahoo.com) , [asadkhan14ch@gmail.com](mailto:asadkhan14ch@gmail.com) , [infoateeqjan@gmail.com](mailto:infoateeqjan@gmail.com) , [nasirdft@gmail.com](mailto:nasirdft@gmail.com)
2. Institute of Basic Medical Sciences, Khyber Medical University, Peshawar, Khyber Pakhtunkhwa, 25120, Pakistan; [ferromind1989@gmail.com](mailto:ferromind1989@gmail.com)
3. Center of Chemical, Pharmaceutical and Food Sciences, Federal University of Pelotas, Pelotas RS, Brazil; [haroongold@gmail.com](mailto:haroongold@gmail.com)
4. Department of Biochemistry, University of Malakand at Chakdara, Dir Lower Khyber Pakhtunkhwa, Pakistan; [mohammadzahoorus@yahoo.com](mailto:mohammadzahoorus@yahoo.com)
5. Department of Pharmacognosy, College of Pharmacy, King Saud University, Riyadh 11451, Saudi Arabia. Email: [rullah@ksu.edu.sa](mailto:rullah@ksu.edu.sa)
6. Department of Pharmaceutical Chemistry, College of Pharmacy King Saud University Riyadh Saudi Arabia. [abari@ksu.edu.sa](mailto:abari@ksu.edu.sa)
7. Department of Chemistry, University of Liverpool, UK. [m.naveed-umar@liverpool.ac.uk](mailto:m.naveed-umar@liverpool.ac.uk)

*****Correspondence**:** drkhalidchem@yahoo.com & [mohammadzahoorus@yahoo.com](mailto:mohammadzahoorus@yahoo.com)

**Table of Contents**

1. **Figure S1.** 2D and 3D displays of various interactions between RdRp and L1 in RdRp-L1 complex.
2. **Figure S2.** 2D and 3D displays of various interactions between RdRp and L2 in RdRp-L2 complex.
3. **Figure S3.** 2D and 3D displays of various interactions between RdRp and L3 in RdRp-L3 complex.
4. **Figure S4.** 2D and 3D displays of various interactions between RdRp and L5 in RdRp-L5 complex.
5. **Figure S5.** 2D and 3D displays of various interactions between RdRp and favipiravir in RdRp-favipiravir complex.


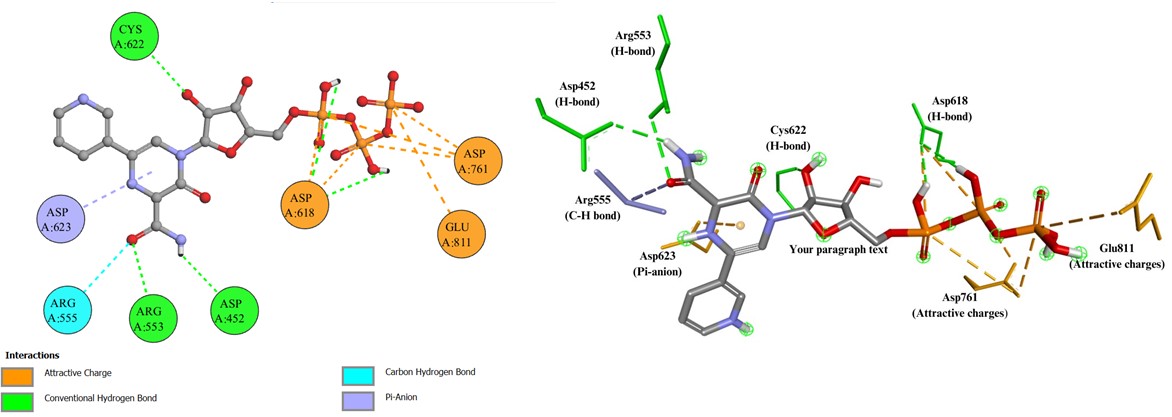


**Figure S1.** 2D and 3D displays of various interactions between RdRp and L1 in RdRp-L1 complex.


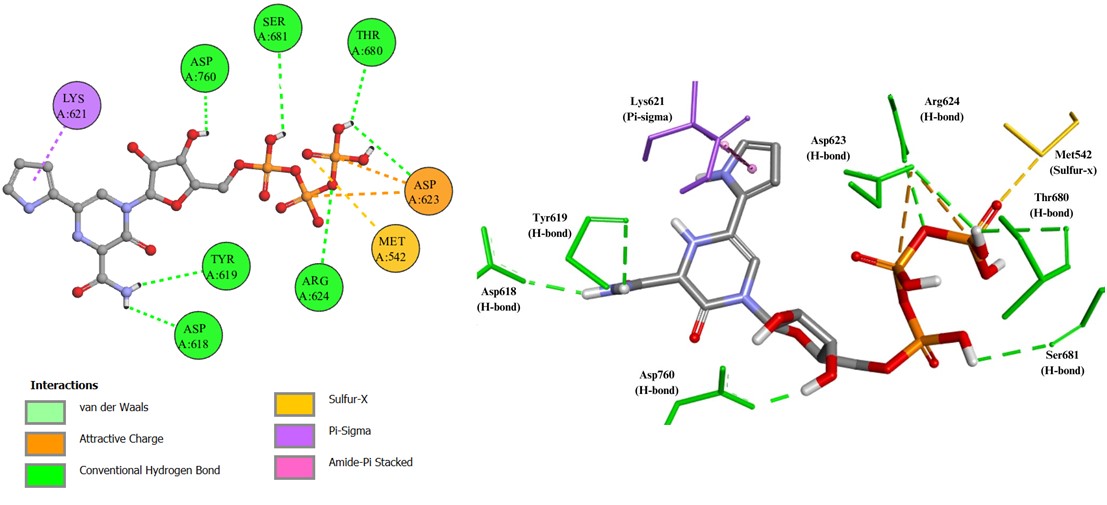


**Figure S2.** 2D and 3D displays of various interactions between RdRp and L2 in RdRp-L2 complex.


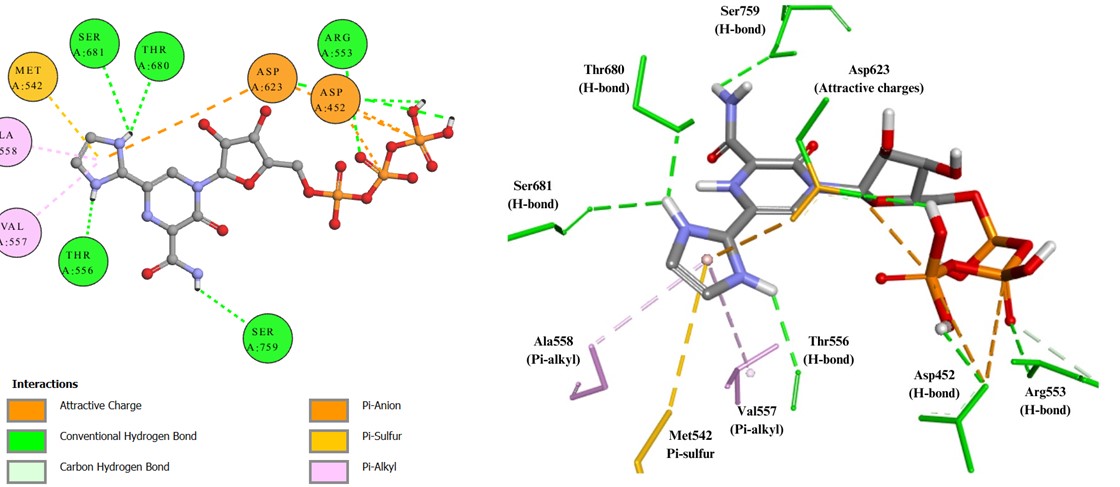


**Figure S3.** 2D and 3D displays of various interactions between RdRp and L3 in RdRp-L3 complex.


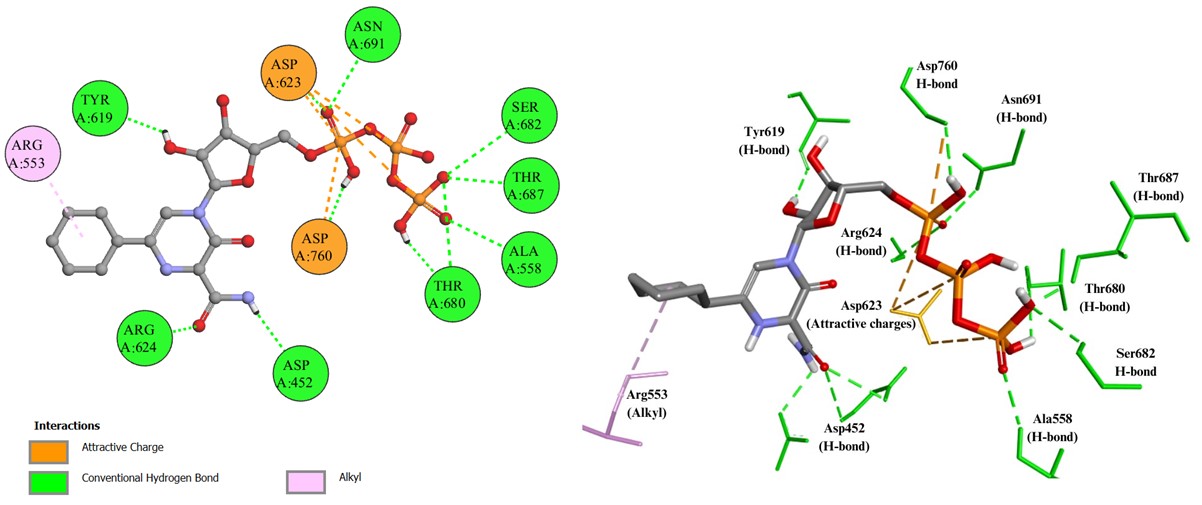


**Figure S4.** 2D and 3D displays of various interactions between RdRp and L5 in RdRp-L5 complex.


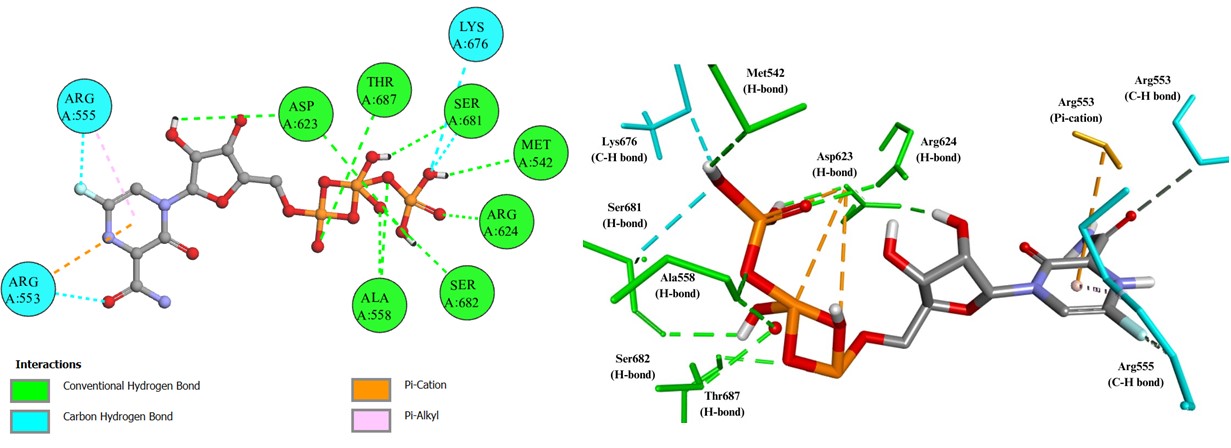


**Figure S5.** 2D and 3D displays of various interactions between RdRp and favipiravir in RdRp-favipiravir complex.

**
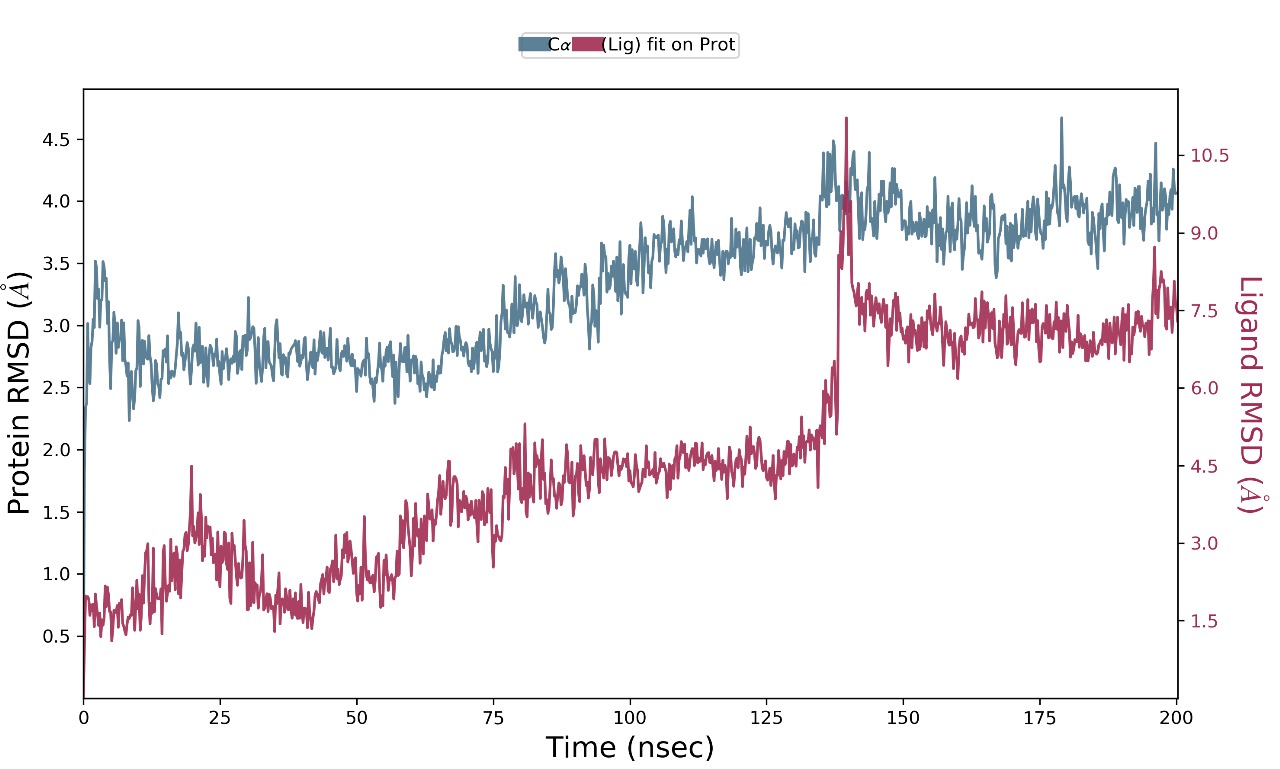
**

**Figure S6.** RMSD curve of parent drug favipiravir F
